# Fungal and Bacterial Pigments: Secondary Metabolites with Wide Applications

**DOI:** 10.3389/fmicb.2017.01113

**Published:** 2017-06-22

**Authors:** Manik Prabhu Narsing Rao, Min Xiao, Wen-Jun Li

**Affiliations:** ^1^State Key Laboratory of Biocontrol and Guangdong Provincial Key Laboratory of Plant Resources, School of Life Sciences, Sun Yat-sen UniversityGuangzhou, China; ^2^Key Laboratory of Biogeography and Bioresource in Arid Land, Xinjiang Institute of Ecology and Geography, Chinese Academy of SciencesÛrúmqi, China

**Keywords:** color, pigments, synthetic dye, microbial pigments, secondary metabolites

## Abstract

The demand for natural colors is increasing day by day due to harmful effects of some synthetic dyes. Bacterial and fungal pigments provide a readily available alternative source of naturally derived pigments. In contrast to other natural pigments, they have enormous advantages including rapid growth, easy processing, and independence of weather conditions. Apart from colorant, bacterial and fungal pigments possess many biological properties such as antioxidant, antimicrobial and anticancer activity. This review outlines different types of pigments. It lists some bacterial and fungal pigments and current bacterial and fungal pigment status and challenges. It also focuses on possible fungal and bacterial pigment applications.

## Introduction

Color affects every bit of life, including the clothes we wear, the furniture in our home, and the allure of food (Downham and Collins, 2000; Manikprabhu and Lingappa, 2013). Just think, for instance, how plants could prepare their own food without chlorophyll or how oxygen could be carried in the body without hemoglobin. It can be said that life on earth depends on pigments (Britton, 1995).

The use of pigments as coloring agents has been practiced since prehistoric times. Archaeologists have uncovered evidence that early humans used paint for aesthetic purposes. The use of pigment in prehistoric times was further proven when pigments and grinding equipments, which were between 350,000 and 400,000 years old, were found in a cave at Twin Rivers, near Lusaka, Zambia (Kassinger, 2003). Pigments were used in different parts of the world. In Europe, it was practiced during the Bronze Age. In China, dyeing with plants, barks, and insects has been traced back more than 5,000 years. In India, it occurred during the Indus Valley period (2500 BC) (Gokhale et al., 2004; Aberoumand, 2011). Henna was used before 2500 BC, while saffron has been mentioned in the Bible (Gulrajani, 2001). In Egypt, mummies have been found wrapped in colored cloth, which showed the presence of alizarin.

The addition of color to food started in Egypt when candy makers added natural extracts to their candy. Similarly, the use of natural colorants in food was seen in Japan in the shosoin text of the Nara period (8th century) that contains references to coloring soybean and adzuki-bean cakes (Aberoumand, 2011).

The first synthetic color, mauvine, was developed by Sir William Henry Perkin in 1856 and this development started a revolution in the history of synthetic colorants (Walford, 1980). Since then, the synthetic color industrial revolution has rapidly proceeded (Downham and Collins, 2000). Synthetic color captured the market due to ease of production, less expensive, no unwanted flavors imparted to food, superior coloring properties, and only tiny amounts are needed to color anything. Sellers at the time offered more than 80 artificial coloring agents. Many color additives at that time had never been tested for their toxicity or other adverse effects, which ultimately led to adverse effects on the health and environment (Downham and Collins, 2000).

Dyes such as tartrazine, cochineal red, and sunset yellow provoke allergies either on their own or in combination with other colorants. Although, some synthetic colorants that had been approved by the Food and Drug Administration (FDA) for use in foods, pharmaceuticals, and cosmetic preparations were later found to promote cancer. Some synthetic dyes have even been withdrawn from external use due to their apparent hazards. For example, benzidine dyes cause bowel cancer, while carbon black (widely used as printing ink pigment) is thought to be a potential carcinogen. From the environmental point of view, unethical discharge of untreated industrial dye effluents produce toxins and persist for long time due to long periods of stability (Babitha, 2009). The drawbacks of synthetic color have increased the global demand for natural pigments (Manikprabhu and Lingappa, 2013).

The main sources for natural pigments are plants or microorganisms. The use of plant pigments has many drawbacks such as non-availability throughout the year and pigment stability and solubility. Large scale plant use may lead to loss of valuable species. For these reasons, the process may not consider viable (Downham and Collins). Microorganisms such as fungi and bacteria provide a readily available alternate source of naturally derived pigments (Arulselvi et al., 2014). Bacterial and fungal pigments have extensive applications (**Table 1**) and have an enormous advantage over plant pigments, including easy and rapid growth in low cost medium, easy processing, and growth that is independent of weather conditions (Manikprabhu and Lingappa, 2013).

**Table 1:** Fungal and bacterial pigments and their applications.

Fungi/Bacteria	Pigment	Application	Reference
**Bacteria**			
*Micromonospora lupine*	Anthraquinone	Antitumor agent	Igarashi et al., 2007
*Streptomyces* sp.	Carotenoid	Food-grade pigment	Dharmaraj et al., 2009
*Chromobacterium*	Violacein	Anti-tumor, anti-microbial, and anti-parasitic agent	Duran et al., 2007
*Chromobacterium* sp. NIIST (MTCC 5522)	Violacein	Antifungal agent	Sasidharan et al., 2015
*Hymenobacter* sp. and *Chryseobacterium* sp.	Carotenoid	Photo-sensitizers in dye sensitized solar cells	Ordenes Aenishanslins et al., 2016
*Streptomyces glaucescens* NEAE-H	Melanin	Anti-cancer agent and anti-oxidant	El-Naggar and El-Ewasy, 2017
*Pseudomonas aeruginosa*	Pyocyanin	Anti-microbial agent	El-Fouly et al., 2015
*Hahella chejuensis*	Prodiginines	Antibiotic	Kim et al., 2007
*Pedobacter*	Carotenoid	Antioxidant	Correa Llanten et al., 2012
*Vogesella indigofera*	Blue pigment	Detect heavy metal	Gu and Cheung, 2001
**Fungi**			
*Aspergillus versicolor*	Asperversin	Antifungal agent	Miao et al., 2012
*Fusarium* sp. JN158	Benzoquinon	Anticancer agent	Zheng et al., 2017
*Fusarium oxysporum*	Anthraquinone	Dyeing of wool fabrics	Nagia and El-Mohamedy, 2007
*Talaromyces verruculosus*	Red pigment	Dye textile having antimicrobial activity	Chadni et al., 2017
*Stemphylium lycopersici*	Anthraquinone	Antioxidant	Li et al., 2017

### Market Trend

There are no reliable published statistics on the size of the color market (Babitha, 2009); however, according to global industry analysts, the demand for organic pigments and dyes is expected to reach almost 10 million tons by 2017. Among the various available pigments, the carotenoids alone are estimated to reach $1.4 billion by 2018 (Venil et al., 2014).

Microbial production of β-carotene costs approximately US$1000/kg versusUS$500/kg for synthetic means. Though microbial pigments are several times more expensive, they still can compete with synthetic dyes for being natural and safe (Venil et al., 2013). There is an increased push to reduce the production costs for microbial pigments by using low cost substrates or strain improvements, and in the near future, there may be a monopoly market for microbial pigments.

Textile industries remains the largest consumer of organic pigments and dyes, while faster growth is expected to occur in other industrial sector such as printing inks, paints, and coating agents. The value of the international food colorant market, which was estimated at around $1.15 billion USD in 2007 (Mapari et al., 2010), may also increase in the future due to food coloring approval for use in the food industry (Aberoumand, 2011).

### Fungal Pigments

Filamentous fungi are known to produce an extraordinary range of pigments such as carotenoids, melanins, flavins, phenazines, quinones, monascins, violacein, and indigo (Dufosse et al., 2014). The use of *Monascus* for ang-kak (red mold rice) production is the oldest recorded use of fungal pigment. *Monascus* produce yellow (ankaflavine, monascine), orange (rubropunctatine, monascorubrine), and purple (rubropunctamine, monascorubramine) pigments which are often encountered in Oriental foods, especially in Southern China, Japan and Southeast Asia. Currently, more than 50 *Monascus* pigments have been identified and studied. More than 50 patents around the globe have been issued concerning the use of *Monascus* pigments in food (Dufosse et al., 2005). *Monascus* pigments possess antimicrobial, anticancer, anti-mutagenic, and anti-obesity properties (Feng et al., 2012).

There are more than 200 fungal species reported for carotenes production (Dufosse et al., 2005). Carotenes production was often found in zygomycetes from the order Mucorales, which includes *Phycomyces*, *Blakeslea*, and *Mucor*. In addition to Mucorales, carotene production has been reported in the basidiomycetes genera such as *Rhodosporidium*, *Sclerotium*, *Sclerotinia*, *Sporidiobolus*, and *Ustilago.* Ascomycetes such as *Aspergillus*, *Cercospora*, *Penicillium*, and *Aschersonia* have also been reported for carotenes production (Avalos and Carmen Limon, 2015).

Pigments such as anthraquinones, naphthaquinones, dihydroxy naphthalene melanin, flavin, anthraquinone, chrysophanol, cynodontin, helminthosporin, tritisporin, and erythroglaucin were reported by genera such as *Eurotium*, *Fusarium Curvularia* and *Drechslera* (Babitha, 2009).

Recent literature extensively has reported the interest in marine organisms with respect to the production of new molecules, including new pigments. Indeed, many marine ecological niches are still unexplored. Marine environments have unique features such as low temperatures, absence of light and high pressure and salinity. These conditions induce marine microorganisms to produce unique substances (Dufosse et al., 2014). Genera such as *Aspergillus* (He et al., 2012), *Penicillium* (Dhale and Vijay Raj, 2009), *Trichoderma* (Blaszczyk et al., 2014), and *Eurotium* (Smetanina et al., 2007) have been reported for pigment production. Marine derived fungal pigments are quite similar to terrestrial derived fungal pigments (Capon et al., 2007); however, some pigments were obtained only from marine fungi. Yellow pigment (anthracene-glycoside asperflavin-ribofuranoside) produced by *Microsporum* sp. appears only in marine-derived fungus (Li et al., 2006).

Several marine-derived endophytic fungi such as *Eurotium rubrum* (Li et al., 2009), *Halorosellinia* (Xia et al., 2007), *Hortaea*, *Phaeotheca*, and *Trimmatostroma* have been reported for pigment production (Dufosse et al., 2014). Apart from plants, marine fungi also make associations with algae and corals. Reports suggest that marine endophytic fungi produce pigments that help to mimic and often increase the beauty of the associated life form (Dufosse et al., 2014). Fungus like *Aspergillus* associates with coral skeleton (*Porites lutea* and *Porites lobata*) and imparts black bands that are quite similar to the coral color (Priess et al., 2000).

Although several fungal pigments have been reported in the literature, they must satisfy several criteria regarding their toxicity, regulatory approval, stability, and capital investment required to bring the products from Petri dish to the market (Malik et al., 2012). Although used for centuries, many microbial pigments are still forbidden in many countries. The best example is the *Monascus* pigment that has been used in Asia for centuries as a food colorant but forbidden in Europe and United States due to the presence of mycotoxin (Dufosse et al., 2005). In this context, methods were developed to avoid toxin productions.

(a) Selection of non-pathogenic strains: to evaluate whether toxin production was strain specific, several strains were screened to check toxicity. The toxin production was observed only in some strains, indicating the toxin production was strain specific.
(b) Through controlling the biosynthesis of the metabolite: toxin production can be controlled through the biosynthesis process; this can be achieved when the metabolic pathway were investigated.
(c) Media selection: researcher observed that the addition or removal of metal ions, carbon sources, and nitrogen sources can affect toxin production (Hajjaj et al., 2000; Dufosse et al., 2005); hence, selection of media plays a crucial role in controlling the toxin production.

Apart from toxin production, microbial pigments should withstand extreme pH and temperature in order to meet industrial standards. Many fungal pigments are stable at a wide pH range.

Pigments produced by *Monascus purpureus, Isaria farinosa, Emericella nidulans, Fusarium verticillioides*, and *Penicillium purpurogenum* showed improved dyeing ability at acidic pH (pH 5) (Velmurugan et al., 2010). Pigment produced by *Thermomyces* was stable from acidic to moderate alkaline conditions (pH 5.1 and 8.0) (Poorniammal and Gunasekaran, 2015). The pigment produced by *Penicillium aculeatum* which is used in soft drink found stable at neutral pH (Mapari et al., 2009). The pigment produced by *Monascus purpureus* was stable even at high alkaline conditions (pH 11) (Huang et al., 2011).

Fungal pigments are stable at various temperatures. Pigments from *Monascus purpureus, Isaria* spp., *Emericella* spp., *Fusarium* spp., and *Penicillium* spp. used for the dyeing pre-tanned leather samples that were found stable at high temperatures (Velmurugan et al., 2010). *Monascus* pigment when added to sausages showed 92% to 98% stability at 4 °C for three months (Fabre et al., 1993).

Though many fungi were reported for non-toxic and stable pigments production, but the development of fermentation derived pigments needs high capital investment in terms of media components. The best example is microbial production of β-carotene. The microbial production of β-carotene cost approximately US$1000/kg versus US$500/kg produced by synthetic means (Venil et al., 2014).

To counter balance the production cost, researchers have shown a great interest in the use of waste or industrial side-streams for the fermentation processes in the development microbial pigments (Panesar et al., 2015). Many fungi were reported for pigment production in low cost substrate. *Monascus ruber* reported for pigment production utilizing corn steep liquor as a nitrogen source instead of yeast extract (Hamano and Kilikian, 2006). Similarly, *Monascus purpureus* produce pigment using grape waste (Silveira et al., 2008). Despite many hurdles, fungal pigments made their way to the market and compete with synthetic colors. Food grade pigments from fungi, including *Monascus* pigments, Arpink red^TM^ from *Penicillium oxalicum*, riboflavin from *Ashbya gossypii*, lycopene, and β-carotene from *Blakeslea trispora* are now available in the market (Dufosse et al., 2014). Many fungal pigments are already used for industrial production, while some are in the development stage (**Table 2**).

**Table 2:** Fungal and bacterial pigments studied or applied for commercial production.

Fungi/Bacteria	Color	Pigment	Status
**Fungi**			
*Monascus* spp.	Yellow	Ankaflavin	Industrial production^#^
*Monascus* spp.	Orange	Rubropunctatin	Industrial production^#^
*Ashbya gossip*	Yellow	Riboflavin	Industrial production^#^
*Cordyceps unilateralis*	Deep blood red	Naphtoquinone	Industrial production^#^
*Monascus* spp.	Red	Monascorubramin	Industrial production^#^
*Penicillium oxalicum*	Red	Anthraquinone	Industrial production^#^
*Blakeslea trispora*	Red	Lycopene	Development stage^∗∗^
*Blakeslea trispora*	Yellow–orange	ß-carotene	Industrial production^∗∗^
*Mucor circinelloides*	Yellow–orange	ß-carotene	Development stage^∗∗^
**Bacteria**			
*Bradyrhizobium* spp.	Orange	Canthaxanthin	Research project^#^
*Streptomyces* sp.	Yellow	Carotenoids	Development stage^∗^
*Streptomyces echinoruber*	Red	Rubrolone	Development stage^∗∗^
*Paracoccus zeaxanthinifaciens*	Yellow	Zeaxanthin	Research project^∗∗^
*Paracoccus carotinifaciens*	Pink–red	Astaxhantin	Research project^∗∗^
*Bradyrhizobium* sp.	Dark-red	Canthaxhantin	Research project^∗∗^
*Pseudomonas* spp.	Blue, green	Pyocyanin	Industrial production^#^
*Flavobacterium* spp.	Yellow	Zeaxanthin	Development stage^#^
*Agrobacterium aurantiacum*	Pink-red	Astaxanthin	Research project^#^

*Data obtained from ^#^Tuli et al., 2015; ^∗^Venil et al., 2013; ^∗∗^Ahmad et al., 2012.*

### Bacterial Pigments

The use of bacteria for pigment production has several advantages over fungi, such as short life cycle and ease for genetic modification (Venil et al., 2013, 2014). However, compared with fungal pigments, most of bacterial pigments are still at the research and development stage (**Table 2**); hence, work on bacterial pigments production should be intensified to make them available on the market. Pigment producing bacteria are ubiquitous and present in various ecological niches, such as soil (Zhu et al., 2007), rhizospheric soil (Peix et al., 2005), desert sand (Liu et al., 2009), fresh water (Asker et al., 2008), and marine samples (Franks et al., 2005). They were reported in low (Nakamura et al., 2003) and high (Manachini et al., 1985) temperature regions, can persist in salt regions (Asker and Ohta, 1999), and even as endophytes (Deng et al., 2011).

Compared with other bacterial groups, the pigment production is more likely to be present in actinobacteria (Marroquin and Zapata, 1954). Various genera such as *Streptomyces, Nocardia, Micromonospora, Thermomonospora, Actinoplanes, Microbispora, Streptosporangium, Actinomadura, Rhodococcus*, and *Kitasatospora* (Rana and Salam, 2014) produce a wide variety of pigments. The genus *Streptomyces* was reported for highest pigment production (Conn and Jean, 1941). Many species of this genus, like *Streptomyces griseus*, *Streptomyces griseoviridis*, *Streptomyces coelicolor* (Darshan and Manonmani, 2015), *Streptomyces cyaneus* (Petinate et al., 1999), *Streptomyces vietnamensis* (Zhu et al., 2007), *Streptomyces peucetius (*Arcamone, 1998), *Streptomyces echinoruber* (Gupta et al., 2011), *Streptomyces shaanxiensis* (Lin et al., 2012), and *Streptomyces caeruleatus* (Zhu et al., 2011) were reported to produce pigments.

Similar to fungi, bacteria also produce a wide range of pigments such as carotenoids, melanin, violacein, prodigiosin, pyocyanin, actinorhodin, and zeaxanthin (Ahmad et al., 2012; Venil et al., 2014).

Two fundamental biotechnological approaches are applied when producing microbial pigments; firstly a search for new sources, and secondly enhancing the yield of already recognized sources either through optimization or strain improvement (Venil et al., 2013). To obtain new sources, several ecological niches were screened, and many pigments producing novel bacterial strains (**Table 3**) were discovered suggesting their vast availability. Strain improvement through chemical and physical mutations significantly varied the pigment production. Strain improvement through ultraviolet (UV) mutation increased prodigiosin production by 2.8-fold when compared with the parent strain (Tao et al., 2005). Employment of UV radiation and ethyl methanesulfonate enhanced pigment production in *Serratia marcescens* (El-Bialy and Abou El-Nour, 2015). Cultural conditions and media optimization showed increased pigment production. *Bacillus* sp. showed significant pigment production when cultivated at pH 7.0 ± 0.1 and a temperature of 34°C (Mondal et al., 2015). Similarly, *Duganella* sp. B2 under optimum pH and nitrogen sources showed increased violacein (4.8-folds) production (Wang et al., 2009).

**Table 3:** List of novel bacteria producing pigments.

Bacteria	Gram	Pigment	Isolated from	Reference
*Paracoccus haeundaensis*	Positive	Orange	Sea water	Lee et al., 2004
*Streptomyces vietnamensis*	Positive	Violet–blue	Soil	Zhu et al., 2007
*Streptomyces shaanxiensis*	Positive	Blue	Sewage irrigation soil	Lin et al., 2012
*Streptomyces caeruleatus*	Positive	Dark blue	Tomato rhizosphere soil	Zhu et al., 2011
*Pseudomonas brassicacearum* subsp. *neoaurantiaca*	Negative	Red–orange	Rhizosphere	Ivanova et al., 2009
*Pseudomonas argentinensis*	Negative	Yellow	Rhizospheric soil	Peix et al., 2005
*Bacillus nakamurai*	Positive	Black	Soil	Dunlap et al., 2016
*Nubsella zeaxanthinifaciens*	Negative	Yellow	Freshwater	Asker et al., 2008
*Kineococcus xinjiangensis*	Positive	Brown	Desert sand	Liu et al., 2009

Recent developments in genetic engineering have made it now possible to modify the bacteria to produce the pigment of interest. *Streptomyces coelicolor*, which produces a blue pigment, can be genetically modified to produce a bright yellow (kalafungin), orange, or yellow–red (anthraquinones) pigment (Bartel et al., 1990; McDaniel et al., 1993).

## Types of Pigments

### Carotenoids

**FIGURE 1:**
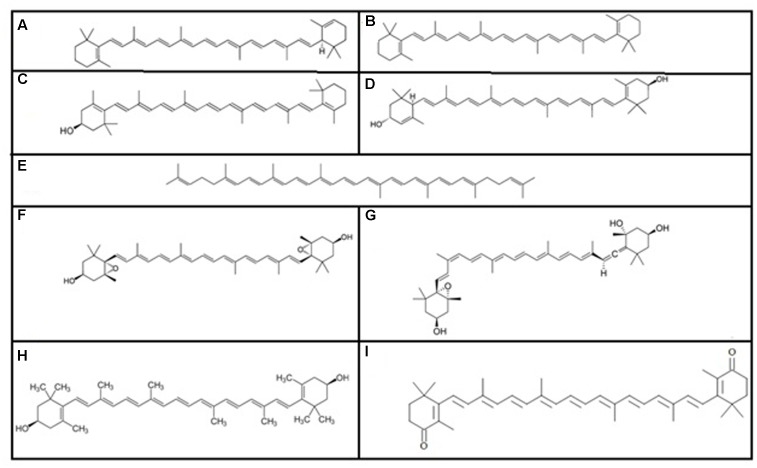
Some important carotenoids: **(A)** alpha carotene, **(B)** beta-carotene, **(C)** cryptoxanthin, **(D)** lutein, **(E)** lycopene, **(F)** violaxanthin, **(G)** neoxanthin, **(H)** zeaxanthin, and **(I)** canthxanthin.

Carotenoids were first isolated by Heinrich Wilhelm Ferdinand Wackenroder (Wackenroder, 1831). All carotenoids are tetraterpenoids (Kocher and Muller, 2011) and there are over 600 known carotenoids, which are divided into two classes: xanthophylls (which contain oxygen) and carotenes (which are purely hydrocarbons, and contain no oxygen). Among the various carotenoids, the most important carotenoids (**Figure 1**) are alpha and beta-carotenes, cryptoxanthin, lutein, lycopene, violaxanthin, neoxanthin, zeaxanthin, and canthxanthin (Rymbai et al., 2011).

Commercial carotenoids are either extracted from vegetables or produced through chemical synthesis. Extraction of carotenoids from plants has many drawbacks such as seasonal and geographic variability while chemical synthesis generates hazardous wastes that can affect the environment. In contrast to these methods, the microbial production of carotenoids shows great praise for use of low-cost substrates and safety (Mata Gomez et al., 2014). Microorganisms producing carotenoids are many and include *Flavobacterium multivorum* (Bhosale and Bernstein, 2004), *Rhodobacter sphaeroides* (Chen et al., 2006), *Rhodotorula mucilaginosa* (Aksu and Eren, 2005), *Sphingomonas* sp. (Silva et al., 2004), *Dunaliella* sp., *Blakeslea trispora*, *Phycomyces blakesleeanus*, *Mucor circinelloides*, *Fusarium sporotrichioides*, *Agrobacterium aurantiacum*, *Paracoccus carotinifaciens, Gordonia jacobea* (Dufosse, 2006), *Sporidobolus salmoncolor, Rhodosporium paludigenum*, and *Rhodotorula glutinis* (Panesar et al., 2015).

Carotenoids producing microorganisms are diverse, isolated from soil (Arulselvi et al., 2014), cave (Liu et al., 2015), marine (Lee et al., 2004), and slattern crystallizer pond (Anton et al., 2002) environments.

The most prominent function of carotenoids is their contribution to harvest light energy. They absorb light and pass the excitation energy onto chlorophyll, thereby extending the wavelength range of harvested light (Kocher and Muller, 2011). They protect chlorophyll from photo damage (Armstrong and Hearst, 1996). They are used as vitamin supplements and play an important role in protection from oxidative stress. Their intake can prevent photo-aging and sun burn (Della Penna and Pogson, 2006). Epidemiological studies have shown that people with high β-carotene intake have a reduced risk of lung cancer (Alija et al., 2004). Carotenoids are used commercially as food colorants, as animal feed supplements, and treatment for obesity. More recently they have been used for nutraceutical, cosmetic, and pharmaceutical purposes (Garrido-Fernandez et al., 2010; Jaswir et al., 2011).

### Melanin

Melanins are indolic polymers (Surwase et al., 2013) classified as eumelanins, pheomelanins, and allomelanins (Banerjee et al., 2014). Melanin is commonly found in all living systems, and their presence in almost every large taxon suggests evolutionary importance (Plonka and Grabacka, 2006).

Melanin production has been reported by a wide variety of microorganisms such as *Colletotrichum lagenarium*, *Magnaporthe grisea*, *Cryptococcus neoformans*, *Paracoccidioides brasiliensis*, *Sporothrix schenckii*, *Aspergillus fumigates* (Langfelder et al., 2003), *Vibrio cholerae, Shewanella colwelliana, Alteromonas nigrifaciens* (Soliev et al., 2011), and many species of the genus *Streptomyces* (Manivasagan et al., 2013).

Melanin confers resistance to UV light by absorbing a broad range of the electromagnetic spectrum and preventing photo-induced damage (Hill, 1992). Melanin is used for mimicry, and protects against high temperatures and chemical stresses. Melanin is extensively used in cosmetics, photo protective creams, eyeglasses, and immobilization of radioactive waste such as uranium. Bacterial melanin genes have been used as reporter genes to screen recombinant bacterial strains. It has anti-HIV properties and is useful for photo voltage generation and fluorescence studies. Melanin is also used to generate monoclonal antibodies for the treatment of human metastatic melanoma (Plonka and Grabacka, 2006; Surwase et al., 2013).

### Prodigiosin

**FIGURE 2:**
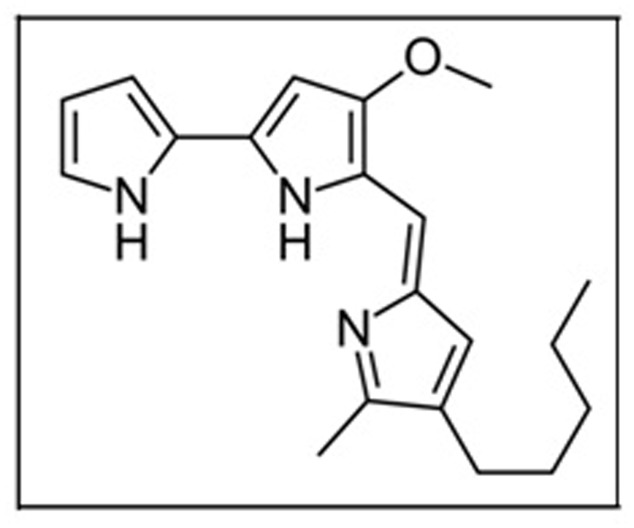
Structure of prodigiosin.

Prodigiosin (**Figure 2**) is a red pigment, first isolated from *Serratia marcescens* (Boger and Patel, 1987).

The name prodigiosin has been attributed to isolation from *Bacillus prodigiosus* which was later renamed as *Serratia marcescens* (Gerber, 1975). Apart from *Serratia marcescens*, prodigiosin production has been reported from *Pseudomonas magneslorubra*, *Vibrio psychroerythrous*, *Vibrio gazogenes*, *Alteromonas rubra*, *Rugamonas rubra*, and *Streptoverticillium rubrireticuli* (Darshan and Manonmani, 2015). Prodigiosin producing microbes are wide spread, and they are isolated from marine samples (Gandhi et al., 1976; Kim et al., 2007), shallow estuarine water (Boric et al., 2011), tidal flat sediment (Yi et al., 2003), and beach sand (Ramaprasad et al., 2015). Prodigiosin acts as a potent therapeutic molecule, especially as an immuno-suppresser and anticancer agents. Prodigiosin also shows insecticidal, antifungal, antibacterial, and anti-malarial activities (Harris et al., 2004; Kamble and Hiwarale, 2012).

### Violacein

Violacein is a violet colored pigment, first described from Gram-negative bacterium *Chromobacterium violaceum* isolated from Amazon River in Brazil. Apart from *Chromobacterium violaceum*, violacein production has been reported from various microorganisms such as *Collimonas* sp., *Duganella* sp., *Janthinobacterium lividum*, *Microbulbifer* sp., *Pseudoalteromonas luteoviolacea*, *Pseudoalteromonas tunicata*, and *Pseudoalteromonas ulvae* inhabiting different environments like soil, marine (Yada et al., 2008; Aranda et al., 2011), glacier (Lu et al., 2009), sea surface (Hakvag et al., 2009), rhizosphere (Aranda et al., 2011), and surface of marine sponge (Yang et al., 2007).

Violacein has been reported for variety of biological activities including antiviral, antibacterial, antiulcerogenic, anti-leishmanial, anticancer, and enzyme modulation properties (Matz et al., 2004; Duran et al., 2007; Soliev et al., 2011)

### Riboflavin

**FIGURE 3:**
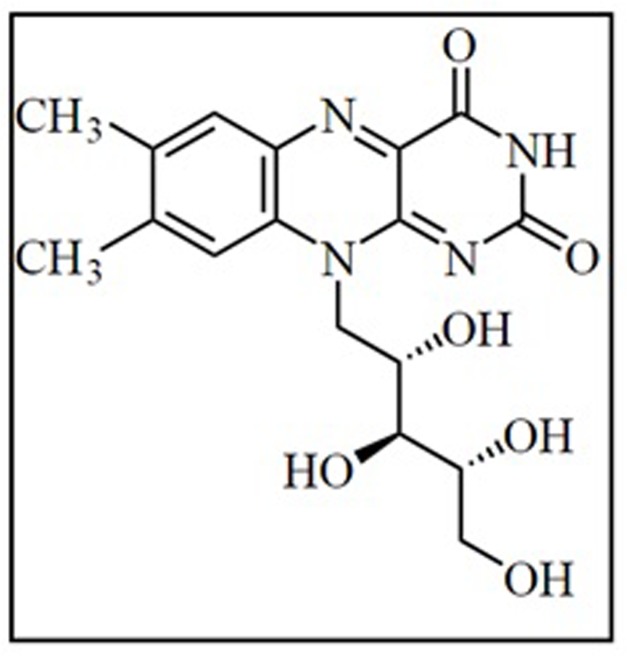
Structure of riboflavin.

Riboflavin (**Figure 3**), also called vitamin B_2_ is water soluble pigment that exhibits a strong yellowish-green fluorescence. It was first isolated by the English chemist Alexander Wynter Blyth (Blyth, 1879). The riboflavin structure was confirmed by Kuhn and Weygand, which suggests that it has two distinct parts consisting of a ribose sugar unit and a three-ring flavin structure known as a lumichrome (Kuhn et al., 1933). Riboflavin is an essential vitamin that needs to be supplemented in the human diet at a concentration of 1.1–1.3 mg per day. Riboflavin acts as a structural component of the coenzymes flavin mononucleotide and flavin adenine dinucleotide. Both coenzymes catalyze non-enzymatic oxidation-reduction reactions by functioning as dehydrogenating hydrogen carriers in the transport system involved in ATP production. For over 30 years, riboflavin supplements have been used as part of the phototherapy treatment for neonatal jaundice. Riboflavin co-treatment with β blockers showed improvement against migraine headaches (Kutsal and Ozbas, 1989; Feroz, 2010). Riboflavin in combination with UV light has been shown to be effective in reducing harmful pathogens found in blood products (Goodrich et al., 2006).

### Pyocyanin

**FIGURE 4:**
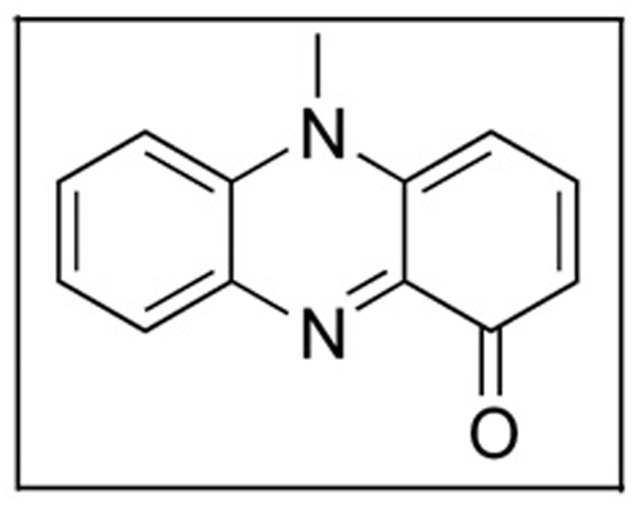
Structure of pyocyanin.

Pyocyanin (**Figure 4**) is a blue pigment produced by *Pseudomonas aeruginosa* (Hassan and Fridovich, 1980). It is composed of two subunits of *N*-methyl-1-hydroxyphenazine (Norman et al., 2004). To synthesize pyocyanin, specific genes must be functional. *MvfR* is a gene which produces a transcription factor which activates *phnAB* genes. These genes produce the molecule quinolone which then regulates operons 1 and 2 of *phzRABCDEFG* which are the key to the synthesis pyocyanin (Mavrodi et al., 2001). Pyocyanin has been used as bio-control agent and possess anti-bacterial and anti-fungal activity (Jayaseelan et al., 2014).

### APPLICATIONS OF PIGMENTS

### Pigments in Textile Industry

The textile industry uses approximately 1.3 million tons of synthetic dyes and dye precursors (Venil et al., 2013). About 200,000 tons of dyes are lost as effluents every year during the dyeing and finishing operations. Unfortunately, most of these dyes escape conventional wastewater treatment processes and persist in the environment as a result of their high stability against light, temperatures, water, detergents, chemicals, soap, and other parameters such as bleach and perspiration (Ogugbue and Sawidis, 2011). In this context, there is a great concern about using eco-friendly dyes. Microbial pigments are eco-friendly colorants applicable to dyeing textile fabrics (Chadni et al., 2017). Many microbial pigments were used to dye different types of fabric. Prodigiosin from *Vibrio* spp. can dye wool, nylon, acrylics, and silk. By using tamarind as a mordant, pigment from *Serratia marcescens* can color up to five types of fabric, including acrylic, polyester microfiber, polyester, silk, and cotton (Yusof, 2008). Anthraquinone from *Fusarium oxysporum* can be used to dye wool fabrics (Nagia and El-Mohamedy, 2007). Recently, Sudha, Gupta and Aggarwal (2016) reported dyeing of wet blue goat nappa skin with the *Penicillium minioluteum* pigment. A red pigment from *Talaromyces verruculosus* shows an adequate color tone for cotton fabric without any cytotoxic effect (Chadni et al., 2017).

Microbial pigments produce different color tones in different textiles. Pigment from *Janthinobacterium lividum* show a bluish-purple color tone on silk, cotton, and wool, while dark blue is seen with nylon and vinylon (Shirata et al., 2000). Similarly, the dyeing ability of yellow pigment from *Thermomyces* was evaluated for cotton, silk, and wool fabrics. It was observed that silk fabric showed high affinity for *Thermomyces* pigments when compared with other fabrics (Poorniammal et al., 2013). Deep blue and red pigments from *Streptomyces* strains NP2 and NP4 also showed significant changes in dyeing ability with respect to the material used. Polyamide and acrylic fibers were stained vibrantly, while cotton and cellulosic fibers were stained weakly (Kramar et al., 2014).

In addition, as a colorant, microbial dyed textiles, showed antimicrobial properties. Textile fabric dyed by prodiginines obtained from *Vibrio* sp. showed antibacterial activity against *Staphylococcus aureus* and *Escherichia coli* (Alihosseini et al., 2008). In the view of the extensive availability of the microbial pigments, their affinity towards different textiles, cost effectiveness, and nontoxic nature, microbial pigments may increase their market appeal and could replace such synthetic colors which are toxic to mankind and nature.

### Pigments as Antimicrobial Agents

The increasing emergence of multidrug resistant bacteria worldwide and the lack of antibiotics to combat such pathogens continue to be a major concern for the medical community (Manikprabhu and Li, 2015). Microbial pigments serve as antimicrobial agents against a wide range of pathogens. Pigments such as carotenoids, melanins, flavins, quinones, monascins, violacein, and indigo have been reported as good antimicrobial agents (Malik et al., 2012). Pigments such as pyocyanin and pyorubin obtained from *Pseudomonas aeruginosa* have shown distinct antibacterial activity against *Citrobacter* sp., which are usually associated with urinary tract and wound infections. Pigments produced from *Micrococcus luteus* KF532949 showed promising antimicrobial activity against wound associated pathogens such as *Staphylococcus* sp., *Klebsiella* sp., and *Pseudomonas* sp. (Umadevi and Krishnaveni, 2013). Pigment obtained from *Streptomyces hygroscopicus*, even showed good antimicrobial activity against drug resistant pathogens such as methicillin and vancomycin resistant strains of *Staphylococcus aureus* and β-lactamase producing strains of *Escherichia coli*, *Pseudomonas aeruginosa*, and *Klebsiella* sp. (Berlanga et al., 2000; Selvameenal et al., 2009). Pigment from *Monascus ruber* showed antimicrobial activity against food borne bacteria (Vendruscolo et al., 2014). Further, inhibition of human pathogenic bacteria such as *Staphylococcus aureus*, *Klebsiella pneumoniae*, and *Vibrio cholera* was observed by the pigment of an endophytic fungal species *Monodictys castaneae* (Visalakchi and Muthumary, 2010).

Efforts in understanding the mechanism of antibacterial activity of some pigments have also been made. The mode of antibacterial action of prodigiosin produced from *Vibrio* sp. DSM 14379 against *Escherichia coli* was evaluated. It was found that the prodigiosin treated *Escherichia coli* cells showed membrane leakage, decreased respiration, and inhibition of protein and RNA synthesis (Danevcic et al., 2016). In view of the above, microbial pigments apart from coloring agents, can be used as novel drugs.

### Pigments as Food Colorants

The development of foods with an attractive appearance is an important goal in the food industry. To make the food appealing, either synthetic or natural colors are added. In recent days, food producers are turning from synthetic to natural colors, due to negative health issues associated with some synthetic colors (Aberoumand, 2011; Venil et al., 2013). Natural colorants from microbes play a significant role as food coloring agents, because of its cheap production, easier extraction, high yield, and no lack of raw materials and seasonal variations (Malik et al., 2012). Many pigments from microbial sources such as red pigment from *Monascus* sp., astaxanthin from *Xanthophyllomyces dendrorhous*, Arpink red^TM^ from *Penicillium oxalicum*, riboflavin from *Ashbya gossypii*, β-carotene from *Blakeslea trispora*, and lycopene from *Erwinia uredovora* and *Fusarium sporotrichioides* were added to the food to increase its appeal (Dharmaraj et al., 2009). Pigment like canthaxanthin used in foods, particularly in products such as cheese, candy, fish, meat, fruits, beverages, snacks, beer, and wine. Pigments like riboflavin (i.e., vitamin B2) are used in beverages, instant desserts and ice creams. Carotenoids can act as a sunscreen to maintain the quality of food by protecting them from intense light (Chattopadhyay et al., 2008).

### Pigments as Antioxidants

An increase in free radicals in the body enhances the chances of occurrence of chronic diseases such as cancer, diabetes, cardiovascular, and autoimmune disorders (Rankovic et al., 2011). To avoid this, antioxidants are used. Antioxidants are molecules that delay or inhibit cellular damage by donating electrons to a rampaging free radical and neutralizing them via their free radical scavenging properties (Lobo et al., 2010). Microbial pigments such as carotenoid, and naphthaquinone demonstrated antioxidant activities (Tuli et al., 2015). Similarly, anthraquinones from the endophytic fungus *Stemphylium lycopersici* (Li et al., 2017) and melanin from *Streptomyces glaucescens* NEAE-H (El-Naggar and El-Ewasy, 2017) were reported as antioxidants. Pigment like xanthomonadin showed antioxidant activity and protection against photo damage (Tuli et al., 2015). Similarly, the antioxidant activity of carotenoid pigment from an antarctic bacterium *Pedobacter* was evaluated. The pigment possessed strong antioxidant capacity and protected the bacterium against oxidative damage (Correa Llanten et al., 2012). The above reports suggest that microbial pigments used as antioxidants may prevent the incidence of many diseases such as cancer and heart disease.

### Pigments as Anticancer Agents

Cancer is one of the most-deadly diseases known to man. The cure for certain types of cancers is considered to be like the Holy Grail since most of the existing treatments are not effective enough to provide full protection (Chakraborty and Rahman, 2012). Efforts to use microbial pigments as anticancer agents have laid the foundation for successful treatments. Many microbial pigments possess anticancer activity. Pigments such as prodigiosin from *Pseudoalteromonas* sp. 1020R have cytotoxicity against U937 leukemia cells (Wang et al., 2012). Melanin from *Streptomyces glaucescens* NEAE-H has been reported for anticancer activity against skin cancer cell line (El-Naggar and El-Ewasy, 2017). Derivatives of anthraquinone from mangrove endophytic fungus *Alternaria* sp. ZJ9-6B has been reported for anti-cancer activity against human breast cancer cell lines (Huang et al., 2011). Pigments obtained from *Monascus* spp. showed remarkable anticancer activity against different cancer cells. Pigments from *Monascus*, such as monascin, showed inhibitory activity against mouse skin carcinogenesis, while ankaflavin showed inhibitory activity against Hep G2 and A549 human cancer cell lines. Similarly, monaphilone A and monaphilone B, exhibits anti-proliferative effect against HEp-2 human laryngeal carcinoma cell lines (Feng et al., 2012). Pigment like prodigiosin has been tested for anticancer activity against more than 60 cancer cell lines and showed a good anticancer activity due to the presence of multiple cellular targets (Darshan and Manonmani, 2015). In the view of the above, microbial pigments can be a potential therapeutic agents to treat cancer.

### Pigments as Bio-indicators

Apart from colorants, antioxidants, antimicrobial agents, and anticancer agents, microbial pigments are used as bio-indicators. Fluorescent pigments from bacteria can be used to check the progress of specific reactions. A key example is phycoerythrin, which is used to predict the rate of peroxy radical scavenging in human plasma. The pigment initially shows fluorescence, however, dark spots appear where the pigment reacts with radicals (Delange and Glazer, 1989).

Pigments are used to detect heavy metals for example, *Vogesella indigofera* produce blue pigment under normal environmental growth condition; however, when exposed to heavy metal like hexavalent chromium, the pigment production did not observed (Gu and Cheung, 2001). Microbial pigments can also be used to monitor temperature variation. *Pantoea agglomerans* produce deep blue pigment only at temperatures of ≥10°C and hence can be used as temperature indicator for the low-temperature-storage management of foods and clinical materials (Fujikawa and Akimoto, 2011).

## Conclusion

Synthetic dyes have caused considerable environmental and health problems. In contrast, microbial pigments are eco-friendly and used in the textile industry, as food colorants, antioxidants, bio-indicators, and antimicrobial and anticancer agents. Though extensive research has been done to bring microbial pigments from the Petri dish to market, still their output cannot fulfill market demand if synthetic dyes withdrawn. Efforts in finding new microbial sources for pigment production and decrease in production cost through optimization, strain improvement and genetic engineering have to be carried out to eradicate toxic synthetic dyes.

## Author Contributions

All authors listed, have made substantial, direct and intellectual contribution to the work, and approved it for publication.

## Conflict of Interest Statement

The authors declare that the research was conducted in the absence of any commercial or financial relationships that could be construed as a potential conflict of interest.
